# A deep learning framework for epileptic seizure detection based on neonatal EEG signals

**DOI:** 10.1038/s41598-022-15830-2

**Published:** 2022-07-29

**Authors:** Artur Gramacki, Jarosław Gramacki

**Affiliations:** 1 Institute of Control and Computation Engineering, University of Zielona Góra, Zielona Góra, Poland; 2 Computer Center, University of Zielona Góra, Zielona Góra, Poland

**Keywords:** Software, Computational science, Epilepsy, Classification and taxonomy, Computational platforms and environments, Data mining, Data processing, Machine learning

## Abstract

Electroencephalogram (EEG) is one of the main diagnostic tests for epilepsy. The detection of epileptic activity is usually performed by a human expert and is based on finding specific patterns in the multi-channel electroencephalogram. This is a difficult and time-consuming task, therefore various attempts are made to automate it using both conventional and Deep Learning (DL) techniques. Unfortunately, authors do not often provide sufficiently detailed and complete information to be able to reproduce their results. Our work is intended to fill this gap. Using a carefully selected 79 neonatal EEG recordings we developed a complete framework for seizure detection using DL approch. We share a ready to use R and Python codes which allow: (a) read raw European Data Format files, (b) read data files containing the seizure annotations made by human experts, (c) extract train, validation and test data, (d) create an appropriate Convolutional Neural Network (CNN) model, (e) train the model, (f) check the quality of the neural classifier, (g) save all learning results.

## Introduction

Epilepsy is a neurological disorder of the brain that affects people of all ages. Around 50 million people worldwide have epilepsy, making it one of the most common neurological diseases globally. It is estimated that up to 70% of people living with epilepsy could live seizure-free if properly diagnosed and treated with the help of anti-epileptic drugs^1^. A relatively small number of patients require surgical intervention (mainly those who are resistant to drug therapy) and/or electrical stimulation^2,3^.

The source of this disease is still not well understood. However, despite this, many patients can be medically treated if seizures are diagnosed on time. As a gold standard, the electroencephalogram (EEG) signal is very important in the diagnosis of epilepsy. The EEG recordings are collected by placing electrodes on the scalp of the patient and then record the electrical signals produced by the brain. Typically, diagnosis using EEG signals is carried out using the knowledge and experience of experts, based on visual inspection of the seizure signals recorded during EEG sessions. However this process is subject to errors, expensive and slow. It is not so rare that two independent experts will evaluate the same electroencephalogram significantly different^4^. This is not a desirable situation, as it may lead to, for example, improper treatment.

The aim of this paper was to develop a complete framework for EEG-based seizure detection using Deep Learning (DL) techniques. We have chosen Convolutional Neural Network (CNN) approach as currently one of the most promising technologies used in the area of data analysis. To present the developed framework we chose the EEG database with carefully selected 79 neonatal EEG recording along with seizure annotations made by three human experts^5^. Let us mention that this dataset was also used by other researchers in their works^6–8^. In^6^ the authors developed a novel method for detecting the nonstationary periodic characteristics of EEG signals to detect periods of seizure and nonseizure activities. In^7^the authors use similar to ours methodology based on CNNs and also note the need for large amounts of training data to achieve satisfactory results. They use a concept called weak annotations^9^ to increase the amount of training data. In^8^ authors assess how different deep learning models and data balancing methods influence learning in neonatal seizure detection. They also propose a model which provides a level of importance to each of the EEG channels which help clinicians understand which channels contributed most to the detection of seizure.

In recent times DL techniques have been shown to be very useful for solving many complex tasks, mainly related to the classification of images, video sequences and text data, see as an example two selected works^10,11^. A lot of papers have also been published in which the authors present the results of numerous automated seizure detection algorithms (SDA) for EEG signals. In three review works^12–14^, the authors have compiled most of these results. Different DL models have been used in SDA such as classical sequential CNNs but also its variants like Convolutional Autoencoders (CNN-AEs), Convolutional Recurrent Reural Networks (CNN-RNNs), Long Short-Term Memory (LSTM)^15^. Our work can be considered as another proposal in this area of research.

It should also be mentioned that there are quite a few non-EEG-based methods for epileptic seizures detection like near-infrared spectroscopy (NIRS), functional MRI (fMRI), positron emission tomography (PET), magnetoencephalography (MEG) or electrocorticography (ECoG)^16^.

### Main contributions of the paper

The main contributions of this paper can be listed as follows:

1. We have proposed a DL-like framework based on CNN for detecting seizure activities and test its usability on a real neonatal EEG dataset.
2. We have proposed a sliding window design to generate fully balanced training data. The design can greatly increase the amount of data which is then fed to the neural network. This can be seen as a kind of data augmentation and this process is crucial for CNNs which typically require large amounts of data to operate effectively and produce useful results.
3. We have developed a solution for reading raw EDF and annotation files with seizure indications made by human experts. Based on these data a training dataset for CNN network is generated and saved in HDF5 format. This work was programmed in R programming environment and shared to the user as ready-to-use R scripts.
4. We have developed a CNN model which can be successfully trained to detect seizure episodes. The obtained results of the classification (at the level of 96–97%) should be considered almost perfect. This work was programmed in Python programming environment and shared to the user as a ready-to-use Python Jupyter notebook.
5. We have made it our priority to ensure that all the presented results are fully reproducible by other researchers. Therefore, all the source codes as well as all the output results obtained by the authors have been included in the Supplementary Information files. Detailed instructions on how to do this have been also included.
  We consider this point particularly important. To cite a very extensive review work^14^, we have that “*...the great majority of papers did not make their code available. Many papers reviewed are thus more difficult to reproduce: the data is not available, the code has not been shared, and the baseline models that were used to compare the performances of the models are either nonexistent or not available.*”.
6. The study will also help readers to analyze their own EEG datasets with only minor modifications to our R and Python codes (adjusting them to possible differences in the EEG data used and in the way seizures are annotated).

**Figure 1:**
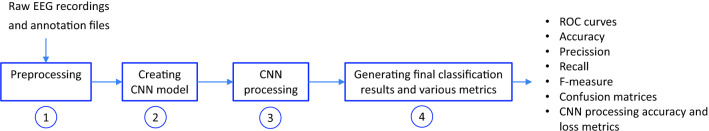
The overall workflow of the of the proposed system.

The overall workflow of the proposed system, schematically depicted in Fig. 1, is decomposed into 4 main phases: (1) preprocessing of the raw EEG recordings and annotation files, (2) building CNN model, (3) training CNN model, (4) generating final classification results. The preprocessing stage is designed to load the input data (raw EDF and annotation files) and convert it to a format that can be submitted to the CNN model. This step has been implemented in the R software version 4.1.2^17^. Building a CNN model, training it and finally generating all the results has been implemented in TesnorFlow version 2.8.0^18^ and delivered as a Python Jupyter notebook^19^.

## Methods

### Cohort

The study was conducted on a carefully selected 79 neonatal EEG recordings dataset. The neonates were admitted to the neonatal intensive care unit (NICU) at the Helsinki University Hospital between 2010 and 2014. The cohort is described in detail in^5^, please refer to the source text. Moreover, the relevant ethics approval has been included therein. All experiments were performed in accordance with the relevant guidelines and regulations.

A neonatal seizure is a seizure in a baby younger than 4 weeks old. Such seizures differ from those of older children and adults, mainly due to brain immaturity^20^. The most frequent neonatal seizures are described as subtle because the clinical manifestations are frequently overlooked^21^.

### Input data

The neonatal EEG dataset consists of (a) 79 raw EDF files, (b) 3 annotation files in CSV and Matlab MAT formats. EDF files contain EEG referential signals recorded with 19 electrodes positioned as per the international 10-20 standard (Fp1, Fp2, F3, F4, F7, F8, Fz, C3, C4, Cz, P3, P4, Pz, T3, T4, T5, T6, O1, O2). Sampling frequency was set to 256 Hz and the signals were recorded in microvolts. The complete dataset is available at https://zenodo.org/record/4940267.

**Figure 2:**
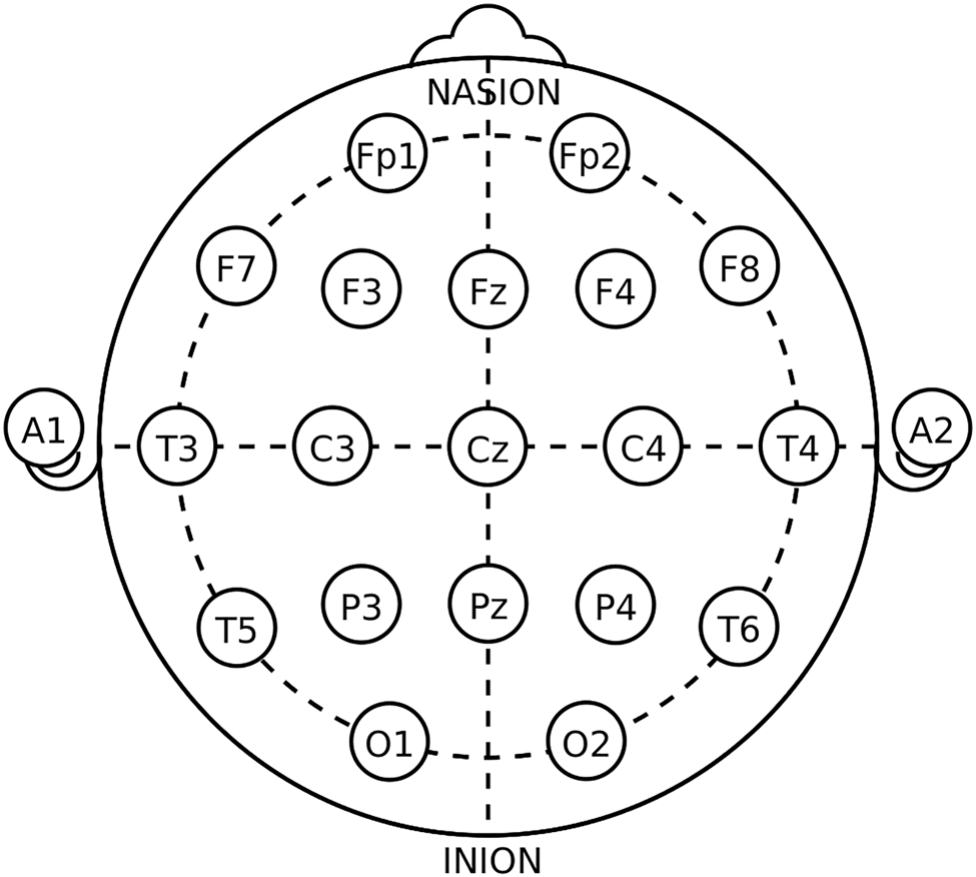
Electrode locations of International 10–20 system for EEG recording (figure taken from^30^).

The raw signals are not used directly. Instead, the so-called bipolar montage was generated known by the slang ’double banana’ (Fp2-F4, F4-C4, C4-P4, P4-O2, Fp1-F3, F3-C3, C3-P3, P3-O1, Fp2-F8, F8-T4, T4-T6, T6-O2, Fp1-F7, F7-T3, T3-T5, T5-O1, Fz-Cz, Cz-Pz), see Fig. 2. This bipolar EEG montage was used by 3 independent experts to annotate the presence of seizures.

The annotation files are sampled with one second resolution. The detailed structure of these files is described in^5^. Since reading this data directly from CSV or MAT files is quite inconvenient, we have collected basic quantitative data on seizures and included them in two tables. Table 6 shows how many seizures were annotated for each infant by each of the three experts. Note that we have 40 neonates with seizures annotated by 3 experts and 17 neonates had seizures annotated by 1 or 2 experts. 22 neonates were seizure free. The experts are marked as A, B or C. Table 7 shows a complete list of lengths of seizures annotated by 3 experts (in whole seconds). The total number of seizures is 1,379, which is obviously the same as shown in the last lines of Table 6. Tables 6 and 7 are very long but the authors decided to include them in its entirety, as obtaining this data from CSV files manually would be very time consuming. The use of an appropriate software here is basically essential. An additional summary of the annotations is provided in Table 8.

Let us note here that in many cases there is a discrepancy in the annotations of individual experts. For example, for infant number 41, experts A and C indicated significantly more seizures than expert B. The lengths of individual seizures also very often vary between experts. Such a variety of end results (no consensus among experts) is rather quite natural in the field of EEG signal analysis^4^.

### Software

The raw EEG recordings were preprocessed (reading and saving in Hierarchical Data Format (HDF5)) using the R software, version 4.1.2^17^. HDF5 format was chosen because it is an ideal choice for storing and organizing large amounts of data.

In our research Keras DL library was used to develop the CNN model^22^. It is also worth to note that Keras is a wrapper to TensorFlow’s framework^18^. Keras was adopted and integrated into TensorFlow in mid-2017. Users can access it via the tf.keras module. TensorFlow, on the other hand, is an open-source DL framework developed by Google and released in 2015. Typically, one can define a model with Keras’ interface, which is easier to use, then drop down into TensorFlow if you need to use a feature that Keras doesn’t have, or you’re looking for a specific TensorFlow functionality.

Due to the required great computing power, our code was run in the Colaboratory cloud service hosted by Google^23^, where fast GPU graphics cards are available (https://colab.research.google.com). The Google Colaboratory service allows users to write and run Python code directly in the WWW browser, which is an extremely convenient solution. A similar functionality is offered by the Kaggle service (https://www.kaggle.com/).

### Data preprocessing

This chapter describes in detail how to prepare datasets for further analysis. This is a very important issue that, if not done properly, may have an impact on the final results of EEG signals classification. Unfortunately, in many papers the authors omit a more detailed description of this stage. We would like to fill this gap here. There are several steps involved in this process, as described below.

**Figure 3:**
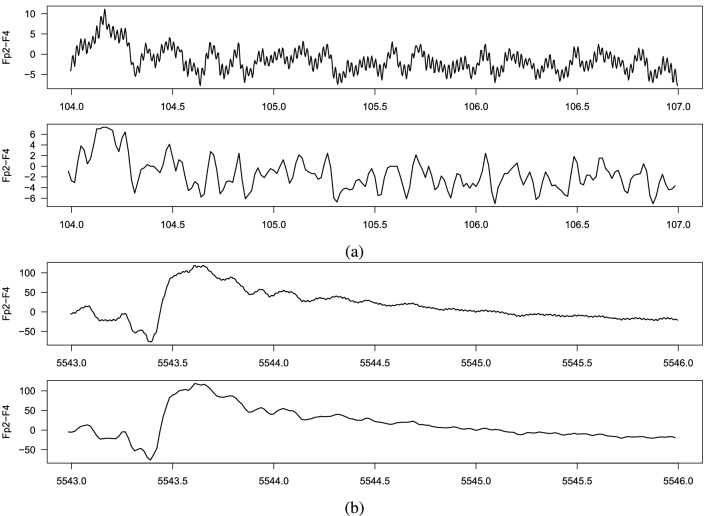
Two exemplary EEG signals. At the top, the original signal sampled at 256 Hz is depicted, at the bottom one can see the signal after reducing the frequency to 64 Hz.

**Figure 4:**
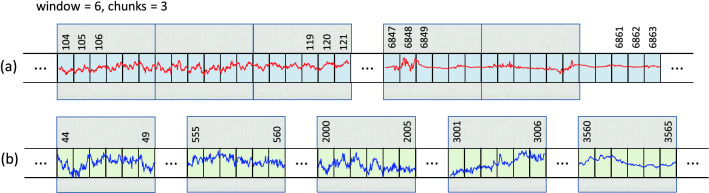
Sliding window design. (**a**) Channel F3-C3 of infant # 1. (**b**) Channel F3-C3 of infant # 10 which is seizures free. Red and blue signals are the real ones. The top signal has 2 seizures annotated by expert A. The first one starts at 104th second and ends at 121st second and is 18 seconds long. The second one starts at 6847th second and ends at 6863rd second and is 17 seconds long (see Table 7). By setting the appropriate values for the window and chunks variables, we can control the length of the samples (window variable) and their total number ( chunks variable). The window length was set to 6 seconds and the number of chunks was set to 3. Note, that the length of the second seizure fragment is 17 seconds. Consequently, it is possible to select only two chunks from the second seizure (although we assumed that we are selecting 3 chunks). From the first seizure one can safely get 3 chunks. The bottom EEG signal has no seizures annotated. We select randomly the same number of chunks (i.e. 5) as we have selected from the top EEG signal. Thanks to this method of selecting chunks, the number of seizure and non-seizure chunks is well balanced. The starting and ending seconds were chosen randomly (form example form 44 to 49 etc.).

**Figure 5:**
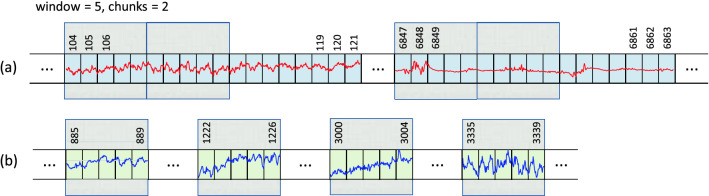
An analogous example to the one shown in Fig. 4. The EEG signals are the same. The figures differ in that window and chunks parameters have different values. Note also that now the window length was set to 5 and 2 chunks from each of the two seizure fragments were selected, see top picture (**a**). Consequently, 4 chunks were selected form the non-seizure signal, see bottom picture (**b**).

**Figure 6:**
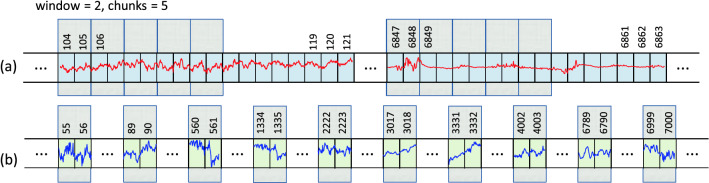
An analogous example to the one shown in Fig. 4. The EEG signals are the same. The figures differ in that window and chunks parameters have different values. Note also that now window length was set to 2 and 5 chunks from each of the two seizure fragments were selected, see top picture (**a**). Consequently, 10 chunks were selected form the non-seizure signal, see bottom picture (**b**).

- *Step 1. Selection of EEG recordings:* The data is analyzed separately for each expert (A, B or C). We are dealing here with a binary classification (seizure / non-seizure). Therefore, it is necessary to select from the available EEG signals those that were assessed by experts as seizures and those assessed as seizure free.
  40 neonates had a seizure annotated by all 3 experts (infants No. 1, 4, 5, 7, 9, 11, 13, 14, 15, 16, 17, 19, 20, 21, 22, 25, 31, 34, 36, 38, 39, 40, 41, 44, 47, 50, 51, 52, 62, 63, 66, 67, 69, 71, 73, 75, 76, 77, 78, 79). We mark this subset as EXP3. 22 neonates were seizure free (infants No. 3, 10, 18, 27, 28, 29, 30, 32, 35, 37, 42, 45, 48, 49, 53, 55, 57, 58, 59, 60, 70, 72). We mark this subset as EXP0. Finally, the remaining 17 neonates had a seizure annotated by only 1 or 2 experts (infants No. 2, 6, 8, 12, 23, 24, 26, 33, 43, 46, 54, 56, 61, 64, 65, 68, 74). We mark this subset as EXP12. Due to the ambiguity in the expert opinion this subset was excluded from the analysis. Table 6 summarises the three subsets.
- *Step 2. Bipolar montage:* In the next step the bipolar montage was generated as described in “Input data” section. At this point, it should be noted that the order of signals in individual EDF files is different, so it is required to always set them in the same order. It is a small but very important part of data preprocessing. For example in the EDF file of the infant No. 1 the order of raw signals is Fp1, Fp2, F3, F4, C3, C4, P3, P4, O1, O2, F7, F8, T3, T4, T5, T6, Fz, Cz, Pz while in the file of the infant No. 2 the order is Fp1, Fp2, F3, F4, F7, F8, Fz, C3, C4, Cz, T3, T5, T4, T6, P3, P4, Pz, O1, O2. This step has been implemented in R.
- *Step 3. Down-sampling:* Sampling frequency of the EEG recordings was set to 256 Hz. In the case of analyzes using neural networks, this frequency is too high and unnecessarily increases the size of the input data (already quite large). Therefore, the data is down-sampled. After performing various experiments, the authors concluded that the optimal down-sampling coefficient should be 4. This means that signals with a frequency of 64Hz are fed to the input of the neural network. Reducing the frequency can, in a sense, be treated as a form of data smoothing. Figure 3 shows two fragments of EEG recordings, each 3 seconds long. In the upper figure, the signal frequency is 256 Hz and in the lower figure it is down-sampled to 64Hz. The aforementioned smoothing effect is clearly visible. Down-sampling has been implemented in R.
- *Step 4. Sliding window design:* From Table 6 one can calculate that an average of 460 seizures were annotated per expert in the EEG dataset. This number is definitely too small to effectively train neural networks (especially when training convolutional neural networks). Therefore, we used a sliding window technique to increase the amount of data which is then fed to the neural network. The second important task of the proposed sliding window design is to select a balanced number of seizure and non-seizure chunks. The process of preparing training data for CNN consists of two steps: a) selection of positive and b) selection of negative samples from all recorded EEG signals. A positive sample is a chunk/fragment with an annotated seizure, a negative sample is a seizure-free chunk/fragment. The design is illustrated in Figs. 4, 5 and 6.
  In all three figures the F3-C3 channel of infant # 1 is depicted (arbitrary selected by the authors). At the top panel there are two EEG signals with seizures annotated by expert A. The first one begins at the 104th second and ends at the 121st second, the second seizure begins at the 6847th second and ends at the 6863rd second (see Table 7). At the bottom panel there is the F3-C3 channel of infant # 10 which is seizure free with randomly selected appropriate number of chunks (5, 4 and 10, respectively).
  We have two parameters at our disposal (window and chunks). Using them, we can define what the resulted data samples will look like. In Fig. 4 we have given window=6 and chunks=3. This means that we want to choose 3 chunks from every annotated seizure, each 6 seconds long. Note that the second seizure is 17 seconds long, so actually it is possible to select only 2 and not 3 chunks (otherwise, we will fall into a non-seizure area). The first seizure is 18 seconds long, so it is possible to select 3 chunks. In Fig. 5 we have given window=5 and chunks=2 and now the lengths of both seizures allow you to select 2 chunks. In Fig. 6 the window size is set to 2 and the desired number of chunks is 5.
  Next, we need to select a relevant number of seizure-free chunks. The binary classification (seizure/non-seizure) requires that the dataset is well balanced. In the context of classification task, this means that the sizes of seizure and non-seizure samples should be more or less the same. The non-seizure chunks are randomly selected from non-seizure EEG signals (bottom panels in Figs. 4, 5 and 6, patients in the group EXP0, see Table 6). As the result, there is no danger that both subsets will be unbalanced. The total number of non-seizure chunks is 5, 4 and 10 respectively in our examples. window and chunks parameters can of course be set to any integer values, according to your needs.
  The above-described method of selecting windows and chunks has been implemented in R.
  Note also that there are studies in which the authors propose methods that allow for the effective detection of epileptic seizures for imbalanced EEG recordings^24,25^. However, our solution based on the CNN approach requires that the data be fully balanced, hence we use sliding windows design described above. Our design, by definition, guarantees the generation of a fully balanced data set. If unbalanced data were fed to the CNN network, the obtained results (binary classification: seizure / non-seizure) would be less reliable and accurate.
  We also point out a subtle difference. In the field of EEG signal analysis, the term *epoch* is used. EEG epoching is a procedure in which specific time-windows are extracted from the continuous EEG signal. In our approach we use the term window and not the epoch to emphasize a slightly different meaning. We do not divide the entire EEG signal into epochs, but select only the fragments that interest us, which we call windows, please see Figs. 4, 5 and 6 for explanation. Because CNN networks require large amounts of data to function properly (mainly we mean reducing the phenomenon known as overfitting), we also introduce the concept of chunks, which allows us to increase the amount of training data we have in our disposal. Let us also mention that the concept of chunks is somewhat similar to the commonly used *data augmentation*, a powerful technique for mitigating overfitting in computer vision. Note also that some authors propose epoch reduction approach for better accuracy of the model^26,27^ but in our case, this technique is not applicable.
- *Step 5. Saving data in HDF5 format:* After completing all the above steps, we obtain the final matrix where fragments with and without seizures are present. For the case shown in Fig. 4, the size of the matrix will be $19 \times 20$, see illustrative Fig. 8 (the last row is the class indicator, 1 means seizure, 0 means non-seizure). Note also that all 18 channels are analyzed simultaneously. The data in this form is then saved in HDF5 format which is very convenient for storing large files of numeric data in an efficient binary format. The saved HDF5 files are passed as input to the appropriate Python routines that implement CNN learning.
  Note that in reality matrices generated from our real EDF files will be much bigger. After down-sampling our EEG dataset every second is represented by 64 datapoints (see Step 3 above). Therefore, the matrix for the data schematically depicted in Fig. 4 would be $19 \times 3840$. Moreover, when working with real data, matrices will be even many times larger since multiple seizures are marked in EDF files and EEG recordings are often longer than 20 seconds (unlike those shown in the Figs. 4, 5 and 6). Additionally, the seizures annotated last for many seconds (see Table 7) and the window and chunks parameters can actually take values greater than those in the toy example shown above. For example from Table 6 we read that expert A annotated 385 seizures in the subset EXP3. If the parameters have the following values: window=2, chunks=3 and f=64Hz the matrix will have $385 \times 64 \times 2 \times 3 = 147,840$ rows.

**Figure 7:**
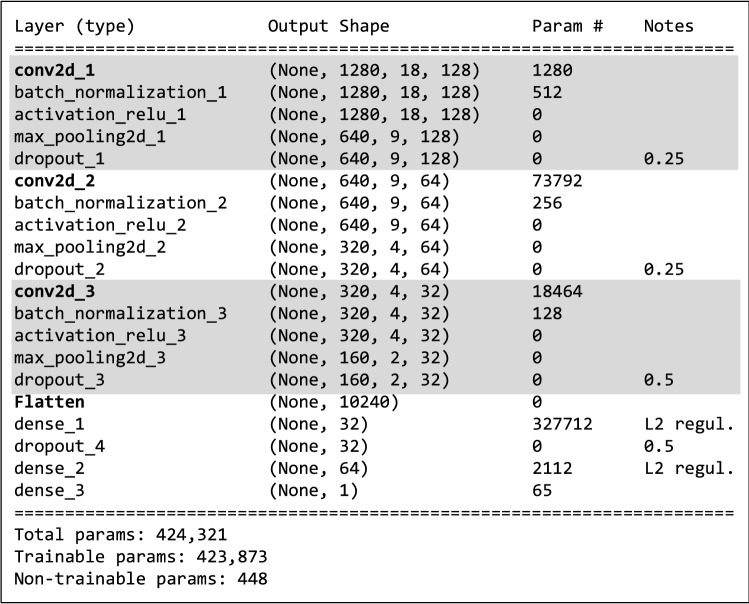
The CNN sequential model used by the authors in all numerical experiments. The Python codes where the model is implemented is available for download in Electronic Supplements.

### Deep learning CNN architecture

The CNN model used in our research has the structure shown schematically in Fig. 7 using summary function implemented in Keras. Its structure is the result of many experiments and tests aimed at developing the most optimal structure possible. Summary of the most important elements of the CNN architecture is depicted in Table 1.

**Table 1:** A summary of the most important parameters of our CNN architecture.

Parameter/Note	Value
CNN network	Sequential, Three Conv2D layers, Two dense layers with L2 regularizers (l2=0.001)
Optimization algorithm	SGD with the parameters: Learning_rate = 0.01, Momentum = 0.5, Nestrrov = False
Activation function	Sigmoid
Loss function	Binary_crossentropy
Batch size	16
Number of epochs	300

### Input data format for CNN

**Table 2:** Description of the content of working subdirectories in the repository attached to the paper.

Directory name	Description
acc_loss	Stores training and validation loss curves side by side, as well as the training and validation accuracy curves
best_models	Stores the best CNN models obtained during training (in terms of model weights, i.e. trainable parameters). The data is saved in the binary HDF5 format. Best models can be loaded later and thus there is no need to train the neural network every time when you want to run a classifier for test data. An example of how to load a best model is shown in the enclosed Jupyter notebook (the load_weights function)
hists	Stores models’ training and validation accuracy and loss values. This data allows you to prepare visualizations of network training, similarly to those depicted in Figs. 13 and 14. The data is saved in the PCKL format (implemented in the Python’s pickle module) and as CSV text files. An example of how to use these files is shown in the enclosed Jupyter notebook (the pickle.load function)
inputs	Stores HDF5 files which are inputs for our CNN model. These files are created in R (EEG_neonatal.R script) using the raw EDF files which are stored in edf directory. To find out exactly which fragments of the original raw EDF files were used in HDF5 files (i.e. the exact samples numbers), files with names beginning with non_seizures_ and seizures_ are additionally generated
logs	Stores log files to be parsed by TensorBoard (TensorBoard is a tool for providing the measurements and visualizations needed during the machine learning workflow).
results	Stores CNN classification results of the validation and test datasets (given in %). The classification results presented in Tables 3, 4 and 5 are the average of the $K=5$ validation scores obtained using K-fold validation scheme. Additionally, execution times for every fold and GPU card types are given
ROC	Stores ROC curves along with the AUC metrics
waveforms	Stores all EEG seizure waveforms annotated by 3 experts. There are 1379 waveforms in total, as depicted in Table 6. The lengths of the waveforms were arbitrarily set at 10 seconds. However, the user can generate waveforms with different lengths by running the generate_eeg_waveforms function in R. See the Supplementary Information files for details how to do this

**Figure 8:**
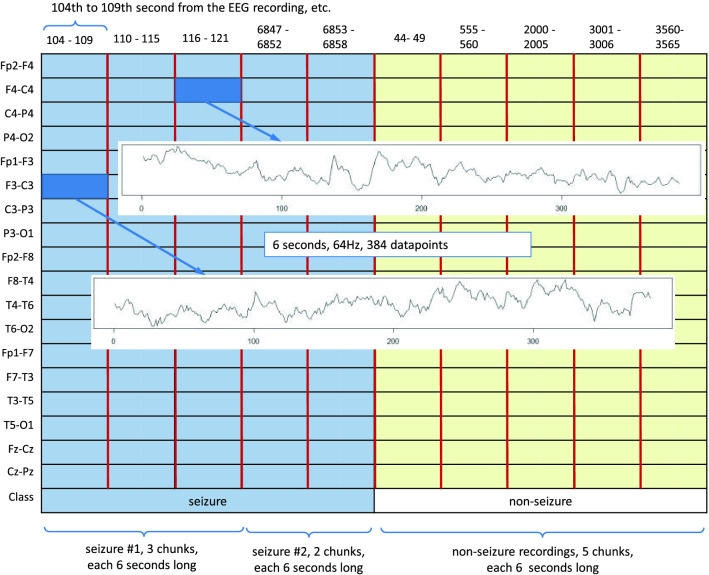
After selecting the desired number of chunks in Fig. 4 one must combine them in a matrix form. In this example the matrix has 18+1 rows (the last row is a class indicator) and 10 columns. Every single cell represents a 6 seconds long EEG signal.

**Figure 9:**
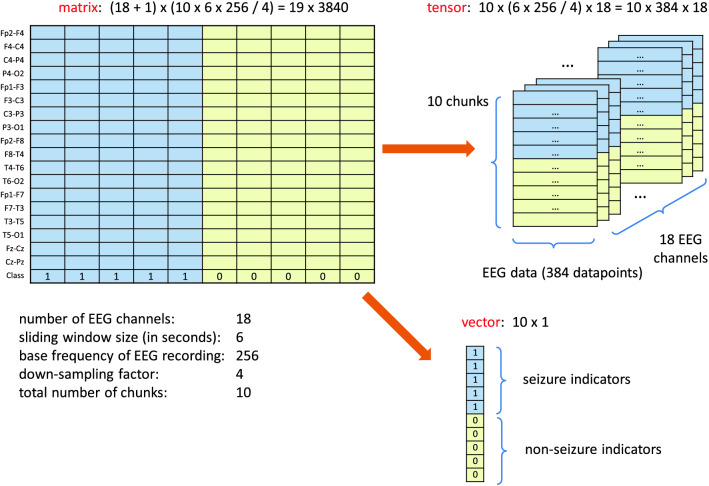
The two-dimensional matrix shown in Fig. 8 cannot be fed into the neural network in this form. In Keras a 3D tensor is required. The figure shows how the 2D matrix must be divided into a tensor and a vector with seizure indicators.

The data stored in the form of two-dimensional matrix shown in Fig. 8 cannot become directly the input for the CNN network implemented in Keras system. It is required to transform it (in other words: rearranging) into the so-called tensors form. Tensor is nothing but a generalization of the concept of a vector or matrix. Only in this form the data can be used in CNN. For those interested, we recommend a very clearly written book^15^. Details of the rearranged matrix are shown in Fig. 9. A tensor of size $10 \times 384 \times 18$ and a vector of size $10 \times 1$ are created. The rearranging has been implemented in Python. Looking at the tensor it is easy to notice how the individual chunks are organized.

**Figure 10:**
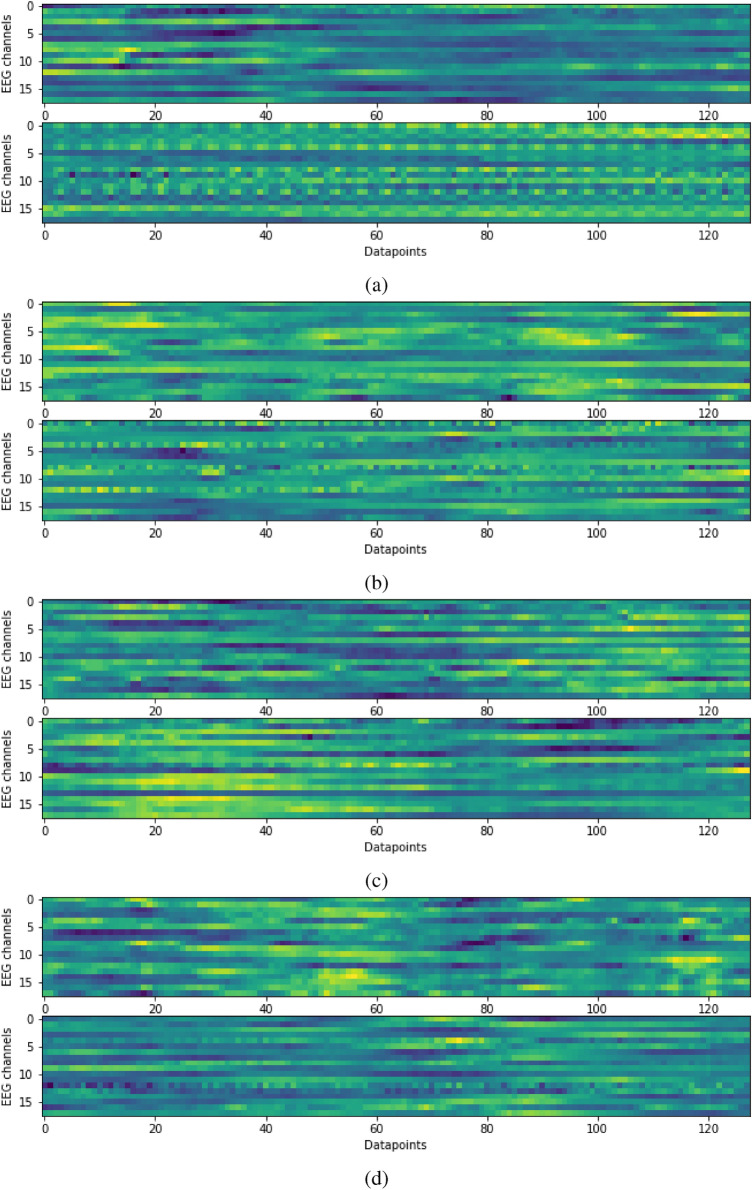
Four exemplary seizure (top) and non-seizure (bottom) fragments of 2 seconds where the sampling frequency is 64 Hz. This gives 128 individual datapoins. The EEG signals are represented as colormaps. It is easy to notice that the analysis of EEG signals, in the form of time series, de facto leads to the analysis of two-dimensional images.

In Fig. 10 four randomly chosen pairs of seizure/non-saizure chunks are depicted. The individual EEG signal values are represented as colormaps. It is easy to notice that the analysis of EEG signals, in the form of time series, de facto leads to the analysis of two-dimensional images. Upper plots show seizure chunks and the lower ones show non-seizure chunks. A certain pattern can be seen in Fig. 10a,b. Top drawings appear more blurry. However, in Fig. 10c,d the human eye cannot see any clear differences. However, very good results of the classification with the use of the CNN approach prove (not for the first time anyway) that deep neural networks learning is able to successfully solve the seemingly unsolvable tasks.

### Training, validation and test data

In order to make CNN working correctly, it is necessary to split the data into three parts: a) training, b) validation and c) test. The model is trained on the training data and its accuracy is constantly checking using the validation data. Once the model is trained, it is tested on the test data. The test set is not involved in the process of building and tuning the model. This is the basic principle that guarantees the objectivity of the obtained results. The splitting data into test and validation sets is usually done randomly. The result of the validation stage will therefore depend on which elements of the dataset will be used during validation and which during the training stages. In this case, the validation result will not be reliable. The best practice in such situations is to use *K-fold cross-validation*. It is based on splitting the available data into K folds (see Fig. 10), creating K identical models and training each of them on K-1 folds. The model is evaluating on the remaining fold. The final validation score is the average of the K validation scores obtained. In our implementation the training and validation subset contains 80% of all data and the test subset contains 20%. The K parameter was set to 5.

The input data for CNN are in the form of tensors and vectors, as shown in Fig. 9. Tensors contain both positive and negative samples and every sample is basically treated completely independently. This is in line with the neural networks principles: the neural network should be provided with as much training data as possible without making any assumptions about the relationship between the training samples. In other words, we do not make any additional assumptions about the acceptance or rejection of a given sample. Each of them is treated the same and it does not matter which patient it comes from.

As an example please refer to Table 6. We can see that expert A annotated 385 seizures in the set named EXP3. Using our sliding design methodology, let’s assume that window=2 and chunks=2. So, we obtain $385 \times 2 \times 3 = 1540$ positive samples in total that will go to the input of the CNN network. Consequently, all available seizure signals are used and no one is left-out.

**Figure 11:**
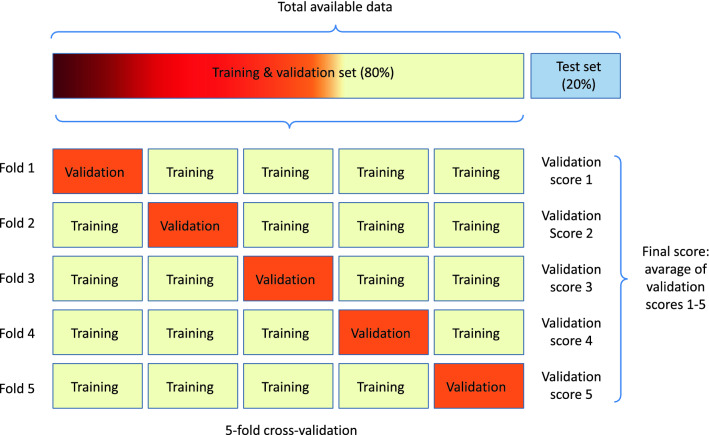
The fivefold cross-validation scheme used in our numerical experiments.

Because neural networks work best when the training data is balanced, therefore, in the next step, we select the same number of negative samples. To make data fully balanced, we select exactly 1540 negative chunks, each with a length of 2 seconds. The negative samples are taken from the patients marked as EXP0 (i.e. without any annotated seizures). Consequently, our CNN network receives 3080 samples. This set is then randomly split into the training-validation part and test part (80% vs. 20%, i.e. 2464 vs. 616 samples). Finally, the training and validation subset is randomly split according to the cross-validation methodology as visualized in Fig. 11. For $K=5$ in every fold $2464 \times 4/5 = 1970$ samples is used for training and $2464 \times 1/5 = 494$ samples is used for validation. The remaining 616 samples are used to evaluate the accuracy of the trained CNN models. The results are summarised in Tables 3, 4 and 5.

**Table 3:** Evaluation results for dataset based on annotations given by expert A. Evaluation was performed on the test set using fivefold cross-validation scheme (see Fig. 11). Three values are given for every window size and every number of contiguous chunks: (a) the test-set accuracy in %, (b) average computation time for fivefolds (see Fig. 11) rounded to full minutes, (c) total number of chunks (see *tensor* in Fig. 9). The given computation times should be treated as indicative as they are very dependent on the instantaneous loads in the Colab system used. 10,000 means that the maximum possible set of contiguous chunks was selected. We can safely set chunks to 10000 and this way we are sure that the maximum possible set of chunks will be selected. Our dataset simply doesn’t have seizures as long as 10,000 seconds.

Window size	Number of contiguous chunks					
	1	2	5	10	20	10000
	Test-set accuracy in %					
1	58.9	70.6	81.2	84.1	86.7	92.7
2	61.5	78.4	83.8	89.5	91.1	95.9
5	68.2	81.4	90.3	92.9	94.7	96.2
10	74.0	79.0	90.0	93.9	96.1	95.6
20	75.4	78.8	88.5	93.1	92.7	94.1

	Average computation time for fivefolds rounded to full minutes					
1	2	4	9	17	42	52
2	3	5	9	10	17	83
5	5	10	27	37	58	51
10	8	16	32	62	73	57
20	13	22	39	55	65	44

	Total number of chunks					
1	781	1,540	3,861	7,699	14,828	90,805
2	781	1,540	3,860	7,386	12,619	45,214
5	781	1,539	3,471	5,833	9,181	17,820
10	780	1,408	2,840	4,474	6,139	8,742
20	650	1,129	2,104	2,897	3,510	4,174

## Results

The seizure annotations presented in^5^ are shared in a specific non-standard format. Therefore, in the first place, we have developed software that allows one to easily load this data and, on the basis of it, prepare batches that can be used as input data for CNN. This part of the software was implemented in the R system. The data generated have the structure shown in Fig. 8. In our experiments we decided to choose the following values for the window and chunks variables: window=[1,2,5,10,20] and chunks=[1,2,5,10,20,10000]. 10000 means that the maximum possible set of contiguous chunks was selected. We can safely set chunks to 10000 and this way we are sure that the maximum possible set of chunks will be selected. Our dataset simply doesn’t have seizures as long as 10,000 seconds. Using these values 30 different datasets were generated for annotations prepared by each of the experts A, B and C. This makes a total of 90 different datasets saved as HDF5 files, see “Replicate the results” section for detailed explanation how to generate these files, how and where they are stored and what their naming convention is.

**Table 4:** Evaluation results for dataset based on annotations given by expert B. The rest of the caption is identical as in Table 3.

Window size	Number of contiguous chunks					
	1	2	5	10	20	10000
	Test-set accuracy in %					
1	63.3	73.0	80.6	82.9	86.2	90.8
2	63.5	75.6	83.8	87.8	91.5	94.3
5	66.7	79.8	88.5	92.3	95.2	96.7
10	66.6	78.9	90.1	94.1	95.7	94.9
20	71.6	78.5	88.5	90.2	93.6	96.0

	Average computation time for fivefolds rounded to full minutes					
1	2	6	14	26	42	67
2	3	6	14	29	46	86
5	6	11	24	42	65	72
10	8	16	32	62	72	56
20	13	24	47	63	80	88

	Total number of chunks					
1	790	1580	3950	7871	15,081	121,393
2	790	1580	3945	7519	13,580	60,526
5	790	1576	3634	6420	10,472	23,979
10	786	1476	3149	5129	6976	11,787
20	690	1257	2419	3325	4256	5701

The CNN learning results are collected in Tables 3, 4 and 5. The best obtained test-set accuracy, the longest computation time and the biggest data size in chunks are printed in bold. We would like to point out here that the obtained results of the classification (at the level of 96%-97%) should be considered very good, almost perfect. It should be emphasized, however, that in order to obtain such results, large amounts of training data are required. For this reason, in principle, a sliding window design was developed and implemented.

**Table 5:** Evaluation results for dataset based on annotations given by expert C. The rest of the caption is identical as in Table 3.

Window size	Number of contiguous chunks					
	1	2	5	10	20	10000
	Test-set accuracy in %					
1	64.2	73.3	82.9	85.6	89.6	93.1
2	61.5	75.8	87.9	90.4	93.2	94.8
5	69.3	84.2	90.9	93.7	96.0	97.0
10	78.5	85.0	91.6	95.4	96.6	96.5
20	80.5	85.0	89.5	93.2	94.7	94.9

	Average computation time for fivefolds rounded to full minutes					
1	3	6	15	30	52	103
2	4	7	17	31	50	65
5	6	12	26	40	87	65
10	10	17	33	61	69	59
20	15	25	45	70	72	46

	Total number of chunks					
1	954	1886	4704	9397	17,541	95,645
2	954	1886	4695	8707	14,738	47,589
5	954	1878	4091	6761	10,452	18,773
10	924	1667	3281	5060	6898	9147
20	743	1272	2362	3236	3812	4312

It is worth noting that the learning process of CNNs is not deterministic. This means that, in principle, we are not able to obtain exactly the same results by performing the same calculations again and again. Each time the results will be slightly different. Nevertheless, the differences will not be too great. All calculations are performed 5 times (fivefold cross validation scheme) and then the average of all partial results is calculated. In Tables 3, 4 and 5 these average results are shown. Nevertheless, all the partial results are included in Electronic Supplements (in the results directory, see the directory structure in “Replication of the results” section).

In the tables we also show average computation time (rounded to full minutes). These results should be treated with a certain distance. A lot depends on the type of GPU card and its temporary load. We worked in the Google Colaboratory and Kaggle cloud environments, where shared resources vary over time and they can vary quickly.

**Table 6:**
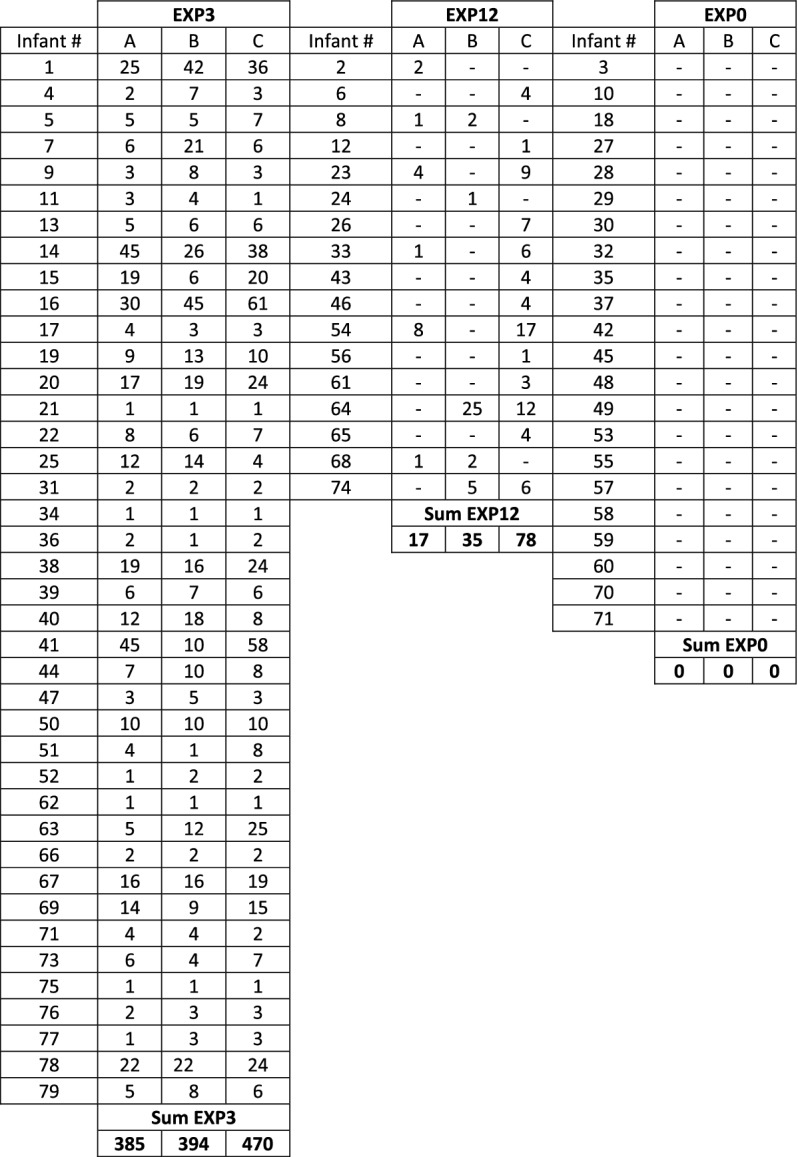
Numbers of seizures for every infant annotated by 3 experts (marked as A, B and C). Cells marked with a hyphen (-) means that no seizure was annotated for a given infant by a given expert. 40 neonates had a seizure annotated by all 3 experts (EXP3 subset), 22 neonates were seizure free (EXP0 subset) and 17 neonates had a seizure annotated by 1 or 2 experts (EXP12 subset).

### Replication of the results

In this section, we provide various details that will help one to replicate all the results of our numerical experiments. We would also like to point out that in some places the source codes are hard-coded with certain elements related to the specificity of the source data used. These are mainly: a) EDF file names, b) number and names of channels stored in EDF files, c) a coding system of seizures annotations. If the provided codes were to be used in the future to analyze a different data set of EEG signals, minor changes would have to be made. The authors declare the necessary help for potential researchers.

**Figure 12:**
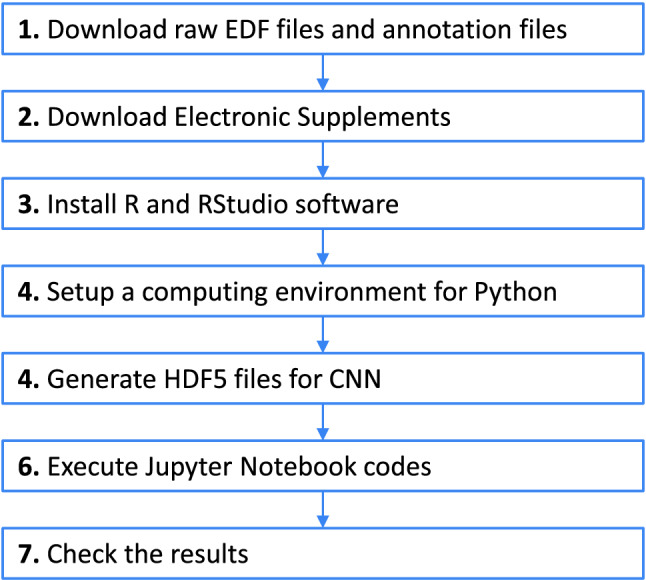
The overall workflow to reproduce the same results that the authors obtained.

The overall workflow to reproduce the results obtained by the authors can be summarised in 7 steps which are shown schematically in Fig. 12.

- *Step 1. Download the dataset of neonatal EEG recordings:* These are available at https://zenodo.org/record/4940267. There are 79 EDF files and 3 CSV annotations files. The EDF files are approximately 4GB in size.
- *Step 2. Download the repository from the Electronic Supplements:* (see “Data and code availability” section). Upload 79 EDF files and 3 CSV files which you downloded in Step 1 to the edf and annotations directories. In the acc_loss, best_models, hists, logs, results, ROC and waveforms directories we have downloaded all our output results. However, you can regenerate these results yourself by running appropriate R and Python scripts, see the next steps below. The complete directory structure is given below and a short description of the content of individual working subdirectories is given in Table 2.
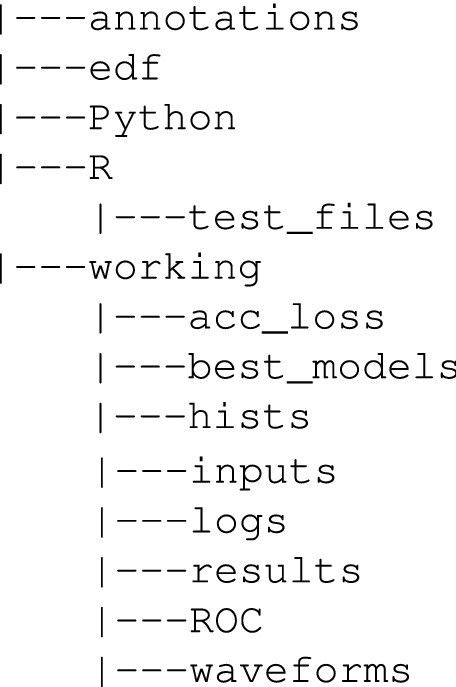
- *Step 3. Install R and RStudio software:* In R install two required packages: *edf* and *rhdf5*. Please note that the latter is installed from the Bioconductor^28^ and not from the primary R package repository.
- *Step 4. Setup a computing environment for Python:* At the beginning, it will probably be most convenient to use cloud-based environment. We may recommend Google Colaboratory (https://colab.research.google.com) or Kaggle (https://www.kaggle.com/). Both environments offer the possibility of using high-performance GPU cards for free. GPU cards are basically necessary to perform the required calculations using CNN. Computing without the use of GPU cards takes many times longer and, in fact, it is unlikely to be completed within a reasonable time.
- *Step 5. Generate required HDF5 files:* To do this run EEG_neonatal.R script (this can take a few hour). Make sure that the current working directory is R. Set also the dir variable to the one indicating the appropriate directory structure in your local computer. The parameters of the generate_samples() function can be changed depending on your actual needs. Those that are saved in the EEG_neonatal.R script will generate exactly the same HDF5 files that were included in the Electronic Supplements. After generating copy all the HDF5 files to the inputs directory. The directory should contain 90 data files ready to fed to the neural network and additionally 184 auxiliary files (these files contain some details about the generated HDF5 file data but you do not need to use them). The data files have the same logical structure as in Fig. 9 and use a uniform naming convention. For example, the file expert_C_5sec_2chunk_64Hz.hdf5 means that data was generated according the annotations made by expert C, the windows size was set to 5 seconds and the number of contiguous chunks was set to 2 (see Figs. 4 and 5). The similar naming convention was used for all other files in the working subdirectories.
  Note: we do not put HDF5 files in the regular Electronic Supplements, as their total size is about 16.6GB. However, for your convenience, we included them in separate zip archives, see “Data and code availability” section.
- *Step 6. CNN processing:* Open the EEG_neonatal.ipynb Jupyter notebook in your favourite Python 3 environment, local or cloud-based. Before the script is run, two global variables, namely WRK_DIR and INPUT_DIR, should be set, indicating the appropriate directories for your runtime environment. Leave the other global variables unchanged if you use input data provided by the authors (i.e. HDF5 files in the working/inputs directory).
  The calculation results will be saved in the subdirectories of the working directory. The files share the same naming convention described above. For example the file: best_model_expert_A_1sec_1chunk_64Hz_fold_0.h5 stores the best model obtained during training of the neural network using the input file expert_A_1sec_1chunk_64Hz.hdf5 during the first fold (fold_0, see Fig. 11. We start counting folds from 0 according to Python convention). To get the complete results presented in the paper, in the __Run__ block set the following values:
  - which_expert=np.array(["A","B","C"]),
  - windows=[1,2,5,10,20],
  - chunks=[1,2,5,10,20,10000].
  In this place, we clearly point out that the calculations will take **several days in total**. It must be realized that calculations performed in the CNN environment, unfortunately, require enormous computing power. The computation time can be reduced five times, but at the cost of leaving the k-fold scheme. Then, in the *Global variables* block set COMPLETE_CALCULATIONS=False. However, the results obtained will be somewhat less objective.
- *Step 7. Inspecting final results:* All final results are stored in the individual subdirectories of the working directory. These are: a) confusion matrices, b) accuracy, precision, recall and F-measure metrics, c) CNN processing accuracy and loss metrics as well as appropriate plots, d) ROC curves.

**Table 7:** Lengths (in whole seconds) of seizures for every infant annotated by 3 experts (marked as A, B and C). When a given expert did not mark any seizures for a given infant, it was marked with a hyphen (-).

Infant	A	B	C
1	18, 135, 59, 29, 31, 49, 57, 23, 87, 23, 31, 93, 104, 34, 25, 24, 78, 122, 74, 333, 19, 23, 99, 15, 17	17, 27, 17, 158, 36, 57, 26, 50, 29, 115, 10, 12, 80, 119, 106, 99, 134, 30, 8, 42, 140, 61, 160, 35, 47, 25, 42, 96, 76, 51, 159, 52, 11, 148, 168, 43, 23, 28, 228, 249, 39, 88	26, 120, 16, 19, 9, 9, 105, 16, 12, 13, 35, 11, 17, 21, 34, 14, 12, 12, 7, 14, 14, 43, 73, 15, 14, 10, 11, 27, 9, 25, 71, 27, 12, 14, 12, 98
2	47, 18	-	-
4	882, 43	48, 30, 33, 931, 42, 52, 25	102, 35, 850
5	127, 631, 534, 853, 1180	107, 621, 454, 825, 1182	147, 620, 457, 571, 245, 419, 725
6	-	-	16, 98, 69, 308
7	16, 18, 133, 141, 168, 149	15, 10, 12, 11, 23, 165, 25, 45, 26, 45, 27, 16, 26, 10, 9, 10, 27, 141, 12, 167, 148	15, 12, 131, 138, 164, 148
8	32	12, 61	-
9	708, 158, 16	12, 715, 26, 157, 37, 52, 17, 25	705, 148, 9
11	21,33, 45	14, 36, 28, 49	40
12	-	-	31
13	292, 496, 112, 233, 138	315, 495, 138, 240, 125, 108	292, 495, 98, 237, 119, 105
14	24, 17, 273, 21, 49, 25, 19, 16, 14, 113, 19, 20, 19, 19, 16, 344, 110, 20, 16, 18, 55, 17, 29, 28, 463, 49, 24, 10, 31, 23, 16, 21, 18, 14, 29, 14, 14, 13, 15, 13, 12, 20, 14, 11, 17	29, 338, 48, 26, 19, 16, 16, 113, 19, 23, 18, 545, 16, 108, 779, 25, 15, 36, 14, 12, 13, 62, 15, 14, 21, 73	61, 24, 337, 48, 25, 18, 16, 15, 26, 85, 19, 20, 17, 16, 378, 136, 15, 114, 29, 29, 461, 52, 24, 9, 65, 15, 19, 14, 11, 20, 13, 11, 12, 45, 13, 13, 19, 70
15	23, 53, 133, 133, 20, 29, 167, 39, 41, 51, 38, 154, 61, 155, 54, 41, 164, 52, 63	164, 64, 51, 155, 93, 46	20, 41, 78, 35, 115, 158, 40, 43, 64, 37, 23, 10, 142, 64, 149, 55, 36, 10, 131, 57
16	45, 14, 19, 18, 42, 29, 14, 21, 18, 17, 14, 13, 16, 24, 12, 15, 22, 11, 17, 25, 13, 32, 17, 26, 24, 25, 22, 23, 23, 9	42, 64, 39, 96, 13, 70, 32, 78, 96, 96, 123, 88, 77, 88, 95, 97, 105, 105, 111, 306, 119, 115, 119, 122, 78, 207, 95, 100, 25, 109, 73, 37, 112, 118, 97, 119, 115, 103, 79, 102, 109, 87, 22, 100, 64	25, 32, 23, 22, 13, 11, 15, 15, 29, 11, 22, 22, 26, 21, 21, 21, 21, 21, 26, 16, 18, 32, 30, 37, 37, 12, 31, 36, 17, 26, 11, 27, 26, 12, 31, 14, 17, 22, 11, 22, 20, 13, 14, 24, 24, 26, 26, 19, 28, 14, 23, 18, 27, 31, 27, 23, 29, 16, 27, 15, 29
17	55, 52, 42, 22	213, 92, 278	40, 9, 11
19	89, 57, 103, 156, 102, 64, 686, 899, 48	81, 112, 92, 18, 108, 155, 163, 79, 686, 15, 1002, 92, 76	63, 46, 61, 158, 140, 63, 674, 901, 30, 46
20	48, 132, 42, 28, 30, 45, 74, 23, 29, 27, 31, 38, 17, 97, 59, 12, 19	193, 225, 22, 59, 219, 103, 81, 167, 140, 75, 58, 66, 92, 116, 17, 41, 10, 76, 34	27, 73, 137, 14, 14, 21, 29, 44, 101, 44, 32, 75, 29, 28, 71, 32, 35, 13, 11, 20, 114, 14, 60, 16
21	43	42	39
22	71, 88, 187, 58, 77, 26, 52, 138	113, 353, 173, 72, 303, 365	57, 83, 150, 66, 97, 53, 134
23	67, 444, 28, 27	-	10, 13, 13, 24, 10, 11, 11, 15, 20
24	-	295	-
25	15, 20, 24, 18, 51, 17, 29, 13, 41, 11, 23, 22	200, 14, 21, 7, 26, 54, 35, 99, 71, 18, 10, 63, 142, 38	13, 23, 20, 17
26	-	-	12, 10, 11, 12, 15, 14, 22
31	80, 102	80, 101	68, 100
33	582	-	13, 11, 9, 15, 14, 27
34	455	452	452
36	147, 345	532	110, 341
38	239, 132, 63, 571, 267, 408, 73, 197, 241, 63, 55, 294, 86, 38, 97, 78, 29, 22, 30	302, 138, 96, 577, 52, 376, 818, 451, 622, 552, 227, 94, 78, 47, 50, 25	235, 100, 53, 269, 159, 91, 49, 292, 396, 73, 197, 38, 236, 56, 88, 228, 53, 34, 70, 70, 20, 15, 27, 15
39	1479, 180, 92, 223, 96, 189	1477, 27, 165, 88, 312, 216, 232	1477, 171, 109, 189, 104, 179
40	29, 19, 22, 20, 17, 29, 100, 21, 26, 33, 135, 150	14, 20, 28, 12, 19, 22, 14, 16, 29, 101, 21, 16, 16, 90, 40, 135, 20, 14	26, 25, 24, 137, 15, 127, 28, 131
41	158, 166, 350, 219, 215, 156, 200, 193, 14, 157, 471, 148, 322, 201, 121, 130, 177, 119, 41, 162, 153, 59, 63, 117, 159, 104, 158, 167, 25, 38, 90, 238, 140, 173, 107, 69, 98, 144, 263, 174, 63, 85, 606, 868, 200	951, 602, 436, 2708, 864, 364, 536, 1232, 1491, 266	159, 167, 131, 197, 213, 211, 85, 83, 211, 187, 11, 58, 130, 169, 94, 138, 147, 172, 164, 216, 153, 134, 38, 51, 79, 145, 51, 163, 152, 168, 160, 158, 117, 171, 170, 43, 41, 14, 83, 243, 148, 195, 140, 145, 47, 93, 154, 210, 45, 189, 59, 74, 32, 633, 380, 479, 206, 36
43	-	-	23, 12, 12, 9
44	20, 82, 18, 102, 15, 19, 85	21, 82, 20, 33, 102, 11, 9, 14, 19, 86	17, 79, 17, 17, 98, 10, 16, 81
46	-	-	36, 25, 10, 12
47	61, 58, 92	63, 67, 35, 58, 89	56, 56, 89
50	89, 98, 91, 97, 94, 104, 97, 102, 70, 88	91, 83, 84, 74, 108, 91, 104, 86, 80, 97	88, 86, 87, 73, 88, 97, 102, 115, 77, 89
51	16, 16, 24, 329	320	56, 20, 12, 29, 11, 33, 23, 307
52	119	53, 47	46, 40
54	167, 238, 136, 55, 114, 371, 110, 76	-	28, 31, 87, 40, 46, 38, 152, 172, 84, 47, 20, 112, 21, 91, 57, 153, 973
56	-	-	814
61	-	-	95, 17, 16
62	382	390	380
63	74, 108, 104, 44, 43	88, 124, 98, 10, 11, 141, 16, 180, 41, 96, 111, 125	49, 339, 204, 56, 13, 101, 106, 68, 36, 172, 169, 68, 123, 82, 25, 102, 27, 17, 24, 13, 11, 117, 11, 20, 87
64	-	35, 27, 58, 43, 62, 48, 47, 48, 75, 97, 80, 83, 86, 50, 40, 59, 88, 131, 75, 128, 56, 81, 71, 57, 80	26, 25, 27, 17, 24, 36, 19, 28, 36, 16, 26, 28
65	-	-	15, 20, 16, 43
66	857, 881	881, 997	823, 725
67	43, 90, 30, 278, 45, 29, 242, 48, 21, 60, 51, 224, 124, 37, 35, 67	43, 137, 29, 307, 60, 46, 252, 54, 28, 59, 63, 216, 137, 62, 81, 74	43, 158, 36, 269, 44, 44, 246, 58, 23, 59, 56, 222, 127, 8, 31, 12, 30, 85, 25
68	33	44, 32	-
69	141, 149, 183, 141, 140, 316, 70, 102, 265, 127, 124, 212, 117, 181	39, 163, 18, 154, 996, 936, 269, 565, 315	40, 149, 13, 146, 183, 232, 142, 309, 71, 150, 148, 265, 571, 121, 176
71	55, 24, 33, 12	72, 48, 82, 117	71, 36
73	25, 235, 26, 266, 24, 292	450, 51, 271, 368	204, 217, 26, 34, 216, 44, 306
74	-	17, 18, 79, 44, 280	11, 89, 52, 16, 20, 15
75	920	925	918
76	46, 431	105, 50, 445	40, 190, 199
77	258	154, 257, 51	108, 256, 156
78	128, 147, 118, 81, 76, 88, 60, 61, 163, 83, 79, 81, 54, 111, 74, 184, 26, 101, 184, 90, 22, 205	124, 143, 119, 23, 82, 75, 75, 45, 84, 160, 86, 52, 78, 183, 141, 89, 184, 116, 190, 124, 152, 188	95, 137, 82, 75, 52, 72, 34, 51, 153, 58, 28, 78, 87, 48, 109, 72, 184, 15, 92, 80, 92, 84, 17, 216
79	43, 19, 56, 26, 54	68, 51, 81, 43, 96, 47, 75, 14	40, 17, 55, 61, 19, 50

**Table 8:** A summary of the seizures annotations by each human expert.

Feature	Expert A	Expert B	Expert C
Number of neonates with seizures annotated	46	45	53
Total seizures annotated	402	429	548
Min, max, mean and median of seizures duration	9 1479 119.3 59.5	7 2708 147,5 79	7 1477 95,8 43
Neonates with seizures annotated by experts A, B, C (consensus annotations)	40		
Neonates with seizures annotated by only one exert	10		
Neonates with seizures annotated by two experts	7		
Neonates where no expert annotated any seizure	22

**Figure 13:**
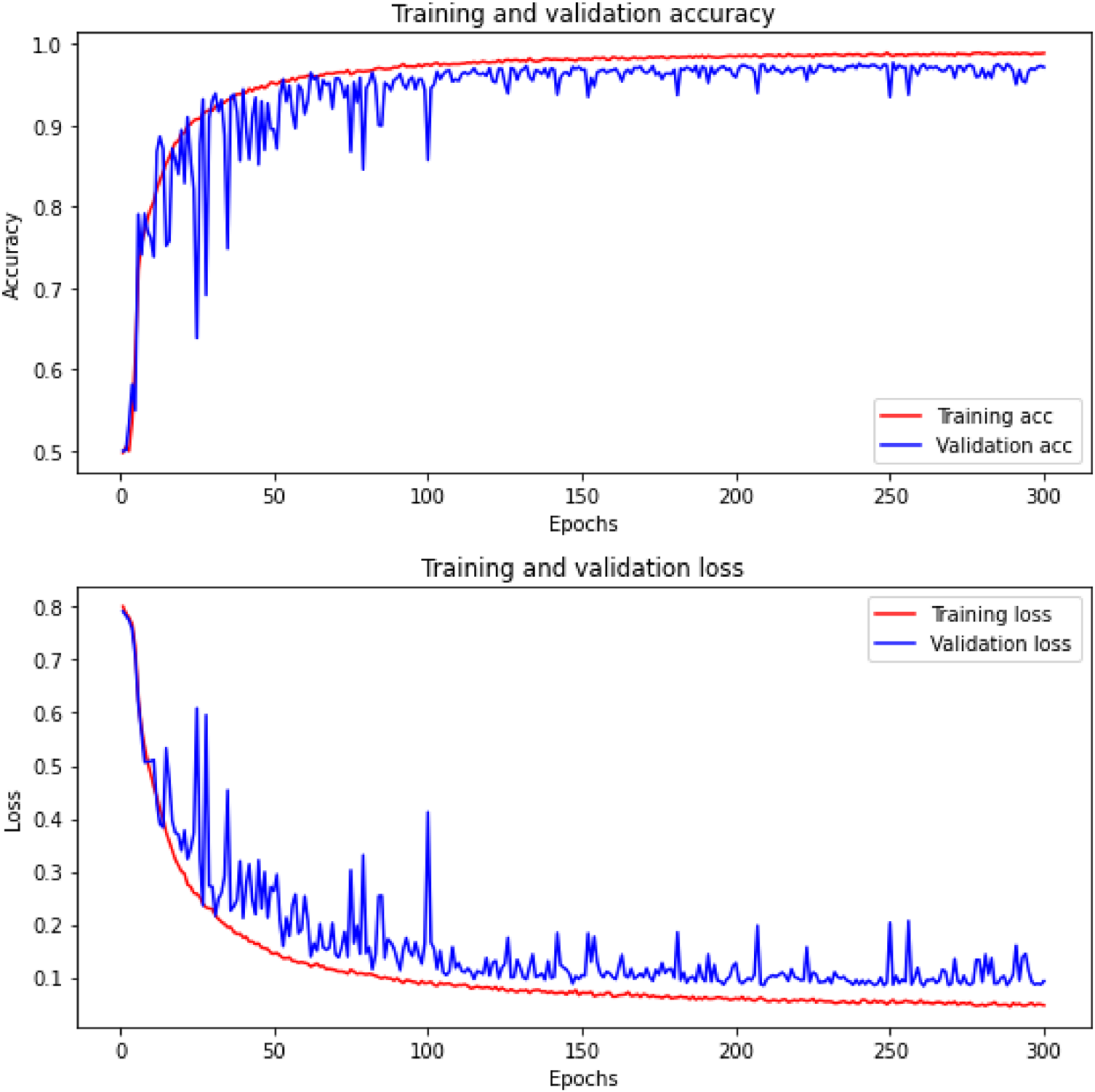
An example of CNN training and validation accuracy (upper curves), as well as the training and validation loss (lower curves). These curves can be considered almost ideal: accuracy is almost 1, loss is almost 0 and there is no very disadvantageous phenomenon called overfitting.

**Figure 14:**
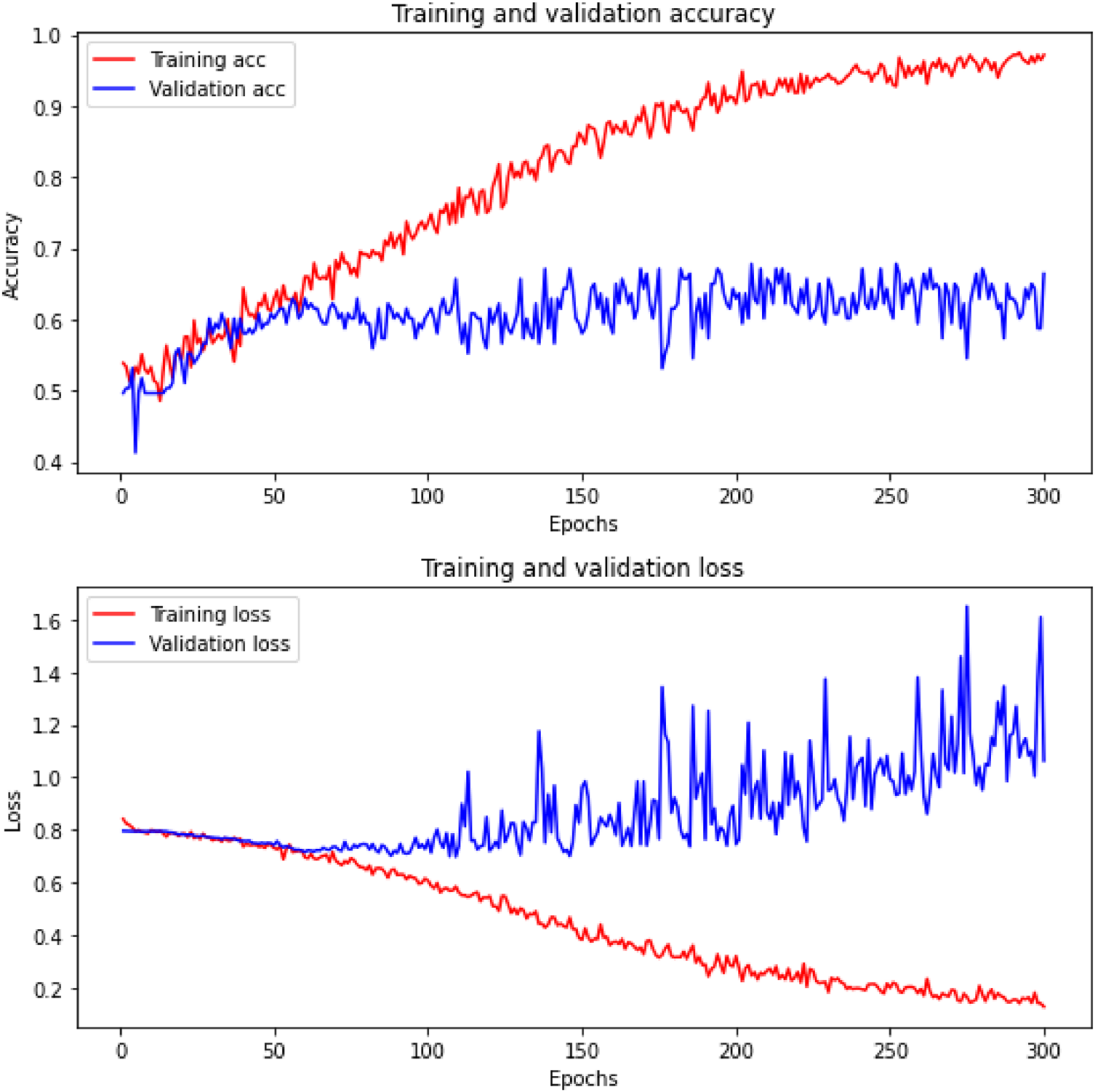
An example of CNN training and validation accuracy (upper curves), as well as the training and validation loss (lower curves). Unlike the curves shown in Fig. 13, these are very bad, overfitting occurs very quickly, in this example around the 50th epoch.

## Discussion

A very thoroughly developed data set was used for our research, although it is a quite specific data set, as it concerns neonates^5^. This dataset have been annotated for neonatal seizures by three independent experts having over 10 years of experience in the visual interpretation of neonatal EEG. So it can be assumed that these results are very reliable. The dataset consists of 79 raw EDF files and 3 CSV files containing the annotations of 3 experts for all the 79 neonates. Times of seizure occurrence are marked by experts with a resolution of one second, i.e. experts indicated in which second a seizure activity started and ended.

A variety of approaches have been proposed to diagnose seizures using EEG recordings. In the days before DL a variety of conventional machine learning algorithms were performed using statistical, time, frequency or time-frequency domains. A comprehensive overview of such methods can be found, among others in the book^29^. The results were better or worse, but the complexity of the EEG signals made it difficult to achieve truly spectacular results. However, it was only the development of DL techniques that resulted in really noticeable progress in the field of automatic seizure detection. We mainly mean DL with the use of convolutional neural networks.

We used sequential CNN model with such regularization techniques as dropout, max-pooling, batch normalization and L2 regularizers. It is important to note that we have developed a fairly flexible method of selecting the number of training samples (through the chunks parameter) and the length of individual samples (in seconds, through the window parameter). The user can thus very easily generate training data having the desired characteristics.

Our research basically confirms that deep neural networks, in order to perform their task well, must be provided with a sufficient amount of training data. The results presented in Tables 3, 4 and 5 show that the total number of training samples is not as important as the length of the individual samples (window parameter). The value window=5 seems to be optimal value. Increasing it does not bring much improvement. As for the chunks parameter, basically the higher its value, the better the results will be. However, keep in mind that the training time of the neural network learning process increases very quickly. The value chunks=20 gives quite good results.

In Fig. 13 we show an example of CNN training and validation accuracy (upper curves), as well as the training and validation loss (lower curves). The dataset was created on the basis of annotations made by expert B with the following parameters: window=5 and chunks=10000. In the context of learning CNNs, these curves can be considered almost ideal: accuracy is almost 1, loss is almost 0 and there is no very disadvantageous phenomenon called overfitting. Note also that in this example the input dataset size is large enough (23,979, see Table 4) that this unfavorable phenomenon does not occur. If, on the other hand, CNN receives too little training data (expert B, window=1 and chunks=1, see Fig. 14), overfitting occurs very quickly, in our example around the 50th epoch.

### Notes on using the framework to analyze datasets other than those used in the article

In our study, we used the neonatal EEG data set, which is basically quite specific. Nevertheless, the proposed framework can also be successfully applied to studies with EEG data from older patients (larger children, adults). In other words, our solution places no restrictions on what kind of patients the EEG data come from. We can consider two cases:

1. Building a new CNN model (or models) based on completely new data.
2. Classification of new data using CNN network already trained by us.

In the first case the main requirement is that seizure annotations be in the same specific format (non-standard in fact) as our data. The annotations must be stored in a CSV file where each column corresponds to a subject (patient) and each row is the annotation of one second of the EEG recording (1 for seizure and 0 for nonseizure. Please study the 3 files in the annotations directory for better understanding the files structure). As for raw EDF files, please note that they may have a slightly different structure (different number of channels, different channel names, etc.). So if someone would like to use our codes to analyze their own EDF datasets, they must meet the following requirements. See also the Electronic Supplements for more information.

- EDF files must be readable by the read.edf() function (edf R package).
- We assume that EDF file names have the format like: eeg phrase and consecutive numbers of subjects, like eeg1.edf, eeg2.edf etc. Otherwise, some minor changes are required in the generate_samples() function.
- The EEG channel names are hard-codded in the function generate_montage(). Depending on the current structure of your raw EDF files, this function must be appropriately adapted to this structure.

In the second case one must be aware that our CNN network has been trained on a certain dataset (quite specific) and is ready to recognize a certain type of seizures (i.e. neonatals ones). Therefore, it should not be expected that when we provide completely different data to our pre-trained CNN network (e.g. based on elderly patients), the network will correctly classify the data. Also some technical details on EEG recordings must be considered carefully. In our case, signals from 18 EEG channels connected according to the ’double banana’ montage were fed to the CNN network. When the new data is not analogous, the classification results can be very questionable. Nevertheless, when the new data is compatible (in the sense as stated above), there are no major contraindications to feed them to our pre-trained CNN network. In the Electronic Supplements one can find some examples.

The Python codes are quite universal and the only requirement is to set a few variables in the *Global variables* block in the Jupyter notebok included. We also assume that the input data filenames (given as HDF5 files) are in the format expert_XXX_YYYsec_ZZZchunk_VVVHz.hdf5 where: XXX - any string indicating for example a human expert who annotated seizures, YYY - window size in seconds, ZZZ - number of contiguous chunks, VVV - base frequency in the HDF5 file. Data stored in HDF5 files must conform to the format shown in Fig. 9.

## Supplementary Information

Supplementary Information.

## Data Availability

All data generated and analysed during this study, as well as R and Python source codes, are included in Supplementary Information files.

## References

[1] World Health Organization. Epilepsy (2021). https://www.who.int/en/news-room/fact-sheets/detail/epilepsy, accessed 20-July-2021.

[2] Jette N, Reid AY, Wiebe S (2014). Surgical management of epilepsy. CMAJ.

[3] Echauz, J. *et al.* Monitoring, signal analysis, and control of epileptic seizures: A paradigm in brain research. In *2007 Mediterranean Conference on Control Automation*, 1–6, 10.1109/MED.2007.4433785 (2007).

[4] Stevenson N (2015). Interobserver agreement for neonatal seizure detection using multichannel EEG. Ann. Clin. Transl. Neurol..

[5] Stevenson NJ, Tapani K, Lauronen L, Vanhatalo S (2019). A dataset of neonatal EEG recordings with seizure annotations. Scientific Data.

[6] Tapani, K., Vanhatalo, S. & N.J., S. Time-varying EEG correlations improve automated neonatal seizure detection. *Int. J. Neural Syst.* **29**, 1850030, 10.1142/S0129065718500302 (2019). 10.1142/S0129065718500302 30086662

[7] O’Shea A, Lightbody G, Boylan G, Temko A (2020). Neonatal seizure detection from raw multi-channel EEG using a fully convolutional architecture. Neural Netw..

[8] Isaev, D. Y. *et al.* Attention-based network for weak labels in neonatal seizure detection. In Doshi-Velez, F. *et al.* (eds.) *Proceedings of the 5th Machine Learning for Healthcare Conference*, vol. 126 of *Proceedings of Machine Learning Research*, 479–507 (2020). PMC7521836 32995751

[9] Saab K, Dunnmon J, Ré C, Rubin D, Lee-Messer C (2020). Weak supervision as an efficient approach for automated seizure detection in electroencephalography. NPJ Digit. Med..

[10] Kong W, Jiang B, Fan Q, Zhu L, Wei X (2018). Personal identification based on brain networks of EEG signals. Int. J. Appl. Math. Comput. Sci..

[11] Ciecierski, K. Mathematical methods of signal analysis applied in medical diagnostic. *Int. J. Appl. Math. Comput. Sci.* **30**, 449–462, 10.34768/amcs-2020-0033 (2020).

[12] Shoeibi A (2021). epileptic seizures detection using deep learning techniques: A review. Int. J. Environ. Res. Public Health.

[13] Craik A, He Y, Contreras-Vidal JL (2019). Deep learning for electroencephalogram (EEG) classification tasks: a review. J. Neural Eng..

[14] Roy Y (2019). Deep learning-based electroencephalography analysis: A systematic review. J. Neural Eng..

[15] Chollet, F. *Deep Learning with Python* (Manning, 2017).

[16] Pandarinathan G, Mishra S, Nedumaran AM, Padmanabhan P, Gulyás B (2018). The potential of cognitive neuroimaging: A way forward to the mind-machine interface. Journal of Imaging.

[17] R Core Team. *R: A Language and Environment for Statistical Computing*. R Foundation for Statistical Computing, Vienna, Austria (2020). https://www.R-project.org.

[18] Abadi, M. *et al.* TensorFlow: Large-scale machine learning on heterogeneous systems (2015). Software available from https://www.tensorflow.org.

[19] Kluyver, T. *et al.* Jupyter notebooks - a publishing format for reproducible computational workflows. In Loizides, F. & Scmidt, B. (eds.) *Positioning and Power in Academic Publishing: Players, Agents and Agendas*, 87–90 (IOS Press, 2016).

[20] Pressler, R. *et al.* The ILAE classification of seizures and the epilepsies: Modification for seizures in the neonate. Position paper by the ILAE Task Force on Neonatal Seizures. *Epilepsia* **62**, 615–628, 10.1111/epi.16815 (2021). 10.1111/epi.16815 33522601

[21] Panayiotopoulos, C. P. *The Epilepsies: Seizures, Syndromes and Management*, chap. 5, Neonatal Seizures and Neonatal Syndromes (Oxfordshire (UK): Bladon Medical Publishing, 2005). https://www.ncbi.nlm.nih.gov/books/NBK2599/. 20821848

[22] Chollet, F. *et al.* Keras. https://keras.io (2015).

[23] Bisong, E. *Google Colaboratory*, 59–64 (Apress, 2019). 10.1007/978-1-4842-4470-8_7, Colab system available at https://research.google.com/colaboratory/.

[24] Siddiqui M, Huang X, Morales-Menendez R, Hussain N, Khatoon K (2020). Machine learning based novel cost-sensitive seizure detection classifier for imbalanced EEG data sets. International Journal on Interactive Design and Manufacturing (IJIDeM).

[25] Sun C (2019). Epileptic seizure detection with EEG textural features and imbalanced classification based on EasyEnsemble learning. Int. J. Neural Syst..

[26] Siddiqui M, Islam M, Kabir M (2019). A novel quick seizure detection and localization through brain data mining on ECoG dataset. Neural Comput. Appl..

[27] Siddiqui M, Morales-Menendez R, Huang X, Hussain N (2020). A review of epileptic seizure detection using machine learning classifiers. Brain Informatics.

[28] Bioconductor Team. Bioconductor: Open source software for bioinformatics. https://www.bioconductor.org/.

[29] Sanei, S. & Chambers, J. *EEG Signal Processing* (Wiley, 2007).

[30] Wikipedia. 10-20 system (EEG)—Wikipedia, The Free Encyclopedia (2021). https://en.wikipedia.org/wiki/10-20_system_(EEG), accessed 20-July-2021.

