# Supplementary material for: A deep learning framework for epileptic seizure detection based on neonatal EEG signals
Source: Sci Rep. 2022 Jul 29;12:13010. doi: 10.1038/s41598-022-15830-2 (PMC9338048; doi:10.1038/s41598-022-15830-2)
Supplement: Supplementary file 1 — Supplementary Information. [file 41598_2022_15830_MOESM1_ESM.pdf]

**Supplementary Information for**

*A Deep Learning Framework for Epileptic Seizure Detection based on Neonatal EEG Signals*

by Artur Gramacki and Jarosław Gramacki
Emails: a.gramacki@issi.uz.zgora.pl, j.gramacki@ck.uz.zgora.pl

1. Raw EDF files and CSV annotations files are available at https://zenodo.org/record/4940267.

2. R and Python source files as well as the complete output results obtained by the authors may be downloads by going to the GitHub website available at https://github.com/artur-gramacki/Epileptic-Seizure-Detection.
